# Efficacy of erlotinib in NSCLC harboring rare EGFR extracellular domain mutation (T263P) and common mutations: Case report and literature review

**DOI:** 10.3389/fonc.2022.954026

**Published:** 2022-09-23

**Authors:** Qian Wang, Yong Wang, Xinwei Zhang, Chen Fang, Xiaoying Qian, Yong Li

**Affiliations:** ^1^Department of Medical Oncology, The First Affiliated Hospital of Nanchang University, Nanchang, China; ^2^Medical Innovation Center, The First Affiliated Hospital of Nanchang University, Nanchang, China

**Keywords:** EGFR extracellular domain mutations, exon 7 T263P mutation, tyrosine kinase inhibitors, erlotinib (ELTN), adenocarcinoma of the lung

## Abstract

The epidermal growth factor receptor (EGFR) typically contains an extracellular domain (ECD), a transmembrane (TM) domain, and an intracellular kinase (KD) domain. ECD mutations of EGFR in NSCLC may affect its normal function and intrinsic resistance to tyrosine kinase inhibitors (TKIs) and the effectiveness of drugs for these patients is unsatisfactory. Recently, we found an EGFR T263P mutation located at the ECD, which has never been reported in Chinese non-small cell lung cancer (NSCLC). Hence, we reported that a patient with advanced lung adenocarcinoma harboring the EGFR T263P mutation, L858R mutation and MET amplification was resistant to osimertinib but significantly benefited from erlotinib and capmatinib treatment. This patient achieved a partial response and had progression-free survival (PFS) for more than 19 months. In summary, we are the first researchers to report in detail on a Chinese patient carrying the T263P mutation and summarize all the ECD mutations in NSCLC. We believe this finding will enlighten us to treat patients with EGFR ECD mutations and more patients deserve further study.

## Introduction

Epidermal growth factor receptor (EGFR) tyrosine kinase inhibitors (TKIs) have been proven to be the most effective therapeutic option for advanced EGFR-mutant non-small cell lung cancer (NSCLC) patients (1). Patients with NSCLC harboring common mutations in the EGFR gene, such as L858R, have shown a remarkable response to TKIs (2). EGFR mutations in NSCLC patients mainly occur in the tyrosine kinase (TK) region and rarely in the extracellular domain (ECD) (3). Mutations in the ECD of EGFR have also been successively reported in NSCLC, but little is known about EGFR ECD mutations in lung cancer. EGFR ECD mutations are generally considered to have a poor response to TKIs. Few studies have reported on the treatment of ECD mutations in NSCLC, especially the efficacy of different ECD mutations to TKIs. Thus, we report a patient with lung adenocarcinoma harboring a rare EGFR mutation, EGFR exon 7 T263P, who was resistant to osimertinib but showed astonishing efficacy against erlotinib. Written informed consent was provided by the patient to use case details and the accompanying images for publication.

## Case presentation

**Figure 1:**
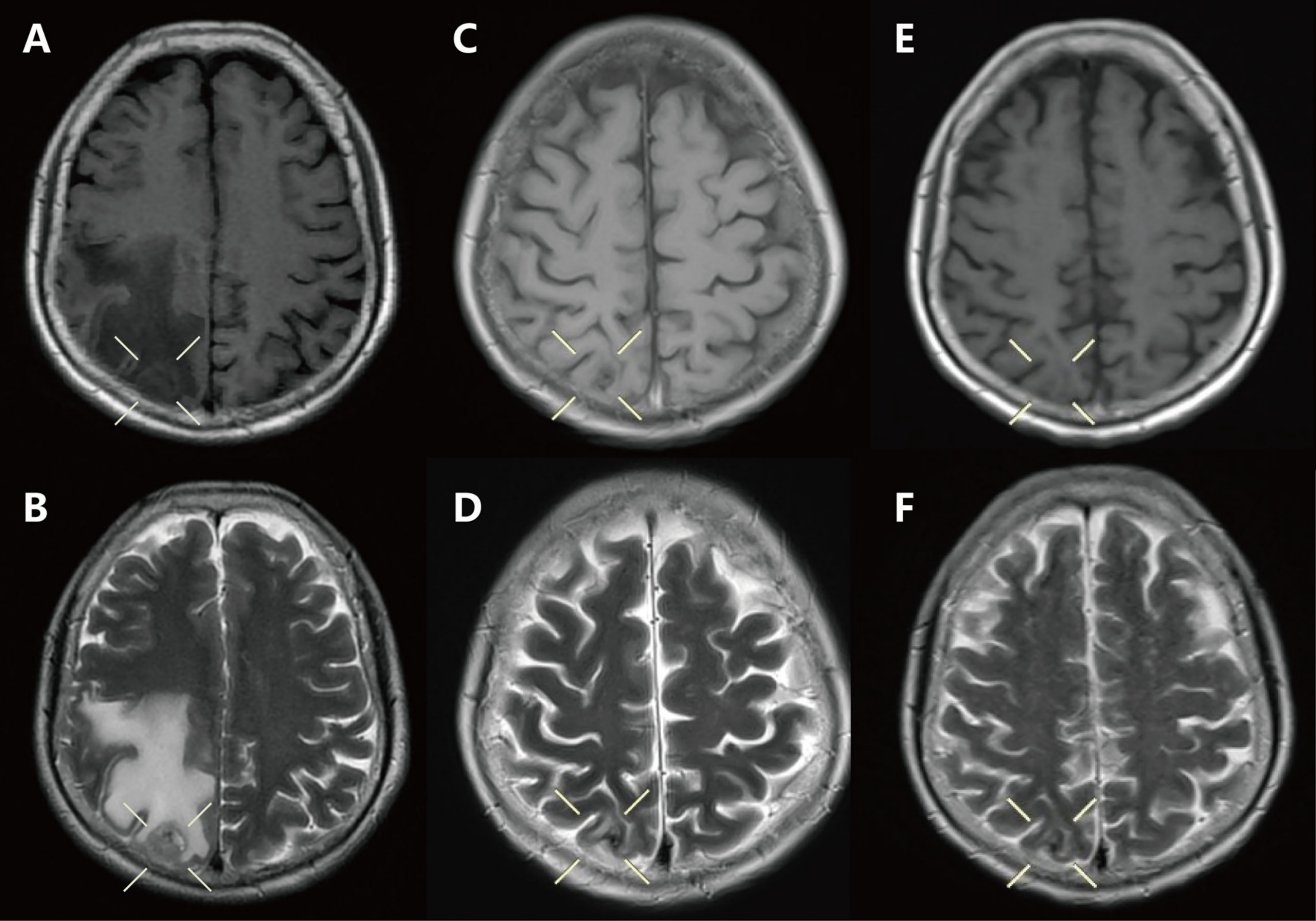
Craniocerebral magnetic resonance imaging of the brain showed: **(A, B)** Baseline: craniocerebral lesion before treatment. **(C, D)** Two months: after brain radiotherapy combined with osimertinib. **(E, F)** Seven months: after the use of erlotinib combined with capmatinib.

**Figure 2:**
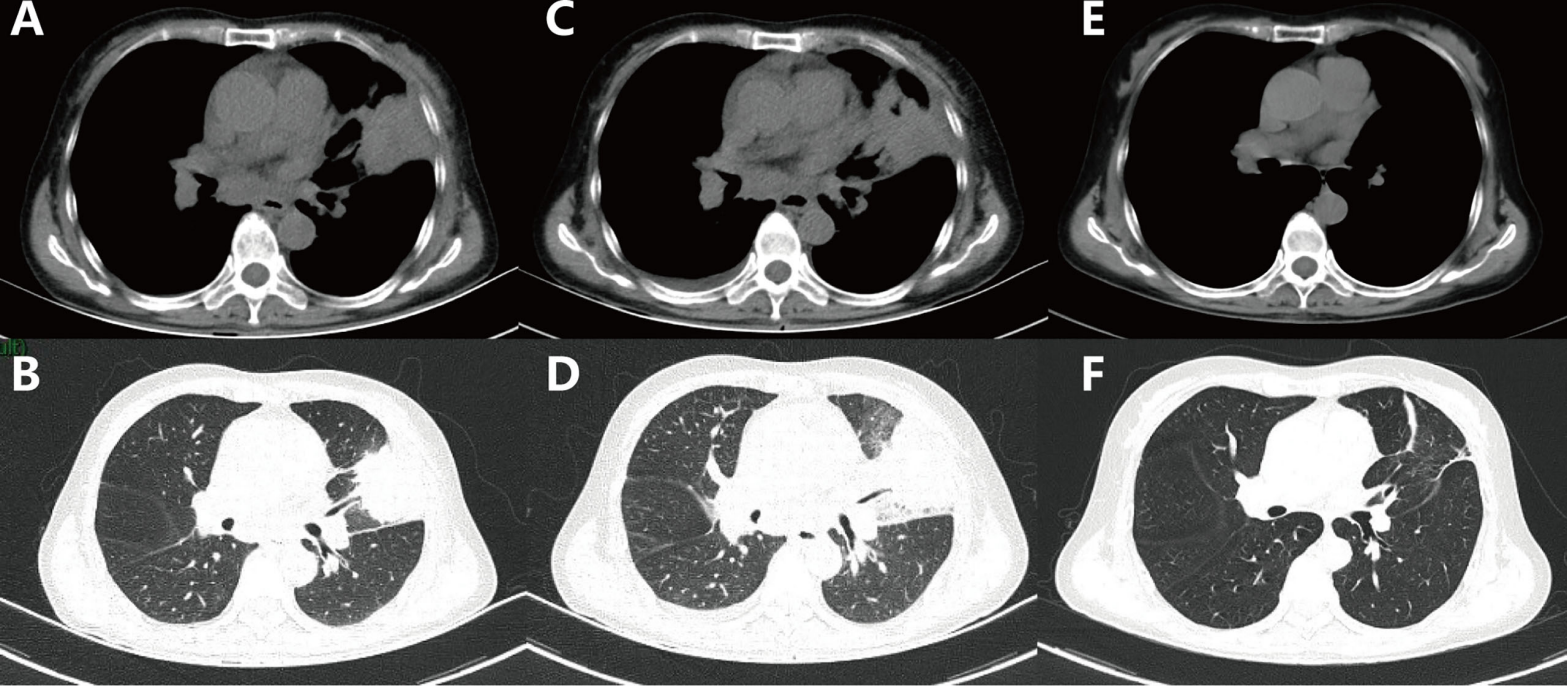
Computed tomography scans of the chest showed the following: **(A, B)** Baseline: lesion in the left lung before treatment. **(C, D)** One month after the use of osimertinib. **(E, F)** Thirteen months after the use of erlotinib combined with capmatinib.

A 61-year-old female was admitted to our hospital with a severe headache in July 2020. Brain magnetic resonance imaging (MRI) showed space-occupying lesions in the right parietal lobe (**Figures 1A, B**). Further evaluation with computed tomography (CT) of the chest and whole-body bone scans revealed a lesion in the left lung (**Figures 2A, B**) and iliac bone metastasis. Fine needle aspiration biopsy of the left supraclavicular lymph nodes showed adenocarcinoma of the lung origin. To identify potentially actionable mutations in the patient, EGFR L858R mutations (allelic fraction, AF=3.37%) in exon 21 were identified by next generation sequencing (NGS, Illumina, NextSeq CN500, Suzhou, China) of the plasma in August 2020. The patient was diagnosed with stage IVB (T1bN2M1) NSCLC, harboring the EGFR the L858R mutation had an Eastern Cooperative Oncology Group performance status (ECOG PS) of 3.

Ten sessions of brain radiotherapy (30 Gy) were performed due to significant headache. At the same time, the patient was administered first-line osimertinib at 80 mg/d. One month later, the patient’s headache was significantly relieved after brain radiotherapy (**Figures 1C, D**) and the ECOG PS of the patient decreased to 1. Nevertheless, a re-examination of chest computed tomography (CT) showed that the lesion in the left upper lobe was enlarged (**Figures 2C, D**). Disease progression (PD) was thus considered. Then, the patient discontinued osimertinib and switched to twice traditional chemotherapy (pemetrexed 700 mg and lobaplatin 40 mg). Meanwhile, lung tumor biopsy was performed for the second genetic testing (Illumina, NextSeq 550Dx, Shanghai, China). In addition to the preserved EGFR L858R mutation, the report revealed a new EGFR exon 7 T263P mutation and MET amplification.

In September 2020, the patient refused chemotherapy for severe adverse effects and started taking erlotinib plus capmatinib. One month later, the patient achieved partial response (PR) with significant tumor shrinkage after erlotinib plus capmatinb treatment. In September 2021, chest CT still showed a PR (**Figures 2E, F**) after 13 months of this treatment. No treatment-related adverse events led to discontinuation. The last follow-up was in February 2022, when the patient remained on the treatment, with a PFS of 19 months and counting (**Figures 1E, F**).

## Discussion

The advent of EGFR-TKIs has made epoch-making sense for treating patients with advanced NSCLC with sensitive EGFR mutations (4). In the present case, the initial detection of the patient’s blood specimen by NGS only revealed the presence of the EGFR L858R mutation. However, the patient had intrinsic resistance to osimertinib. One month later, an EGFR T263P mutation and MET amplification were detected from the patient’s primary tumor samples by the second NGS testing. To understand the actual EGFR mutation before osimertinib treatment, we tried NGS using the archived specimen. Unfortunately, it was not successful due to insufficient tissue in the supraclavicular lymph nodes. Interestingly, a prospective study observed only a 62.0% by variant concordance rate among primary tumor, metastatic lymph nodes, and plasma, suggesting that there is some deviation between the detection of plasma samples and the detection of primary tumor samples (5). Therefore, the genetic test results of the first blood sample do not reflect the actual mutation status of the patient. Besides, the second NGS platform was not limited to common mutations such as EGFR, MET and ALK, but also included rare mutation sites like EGFR extracellular domains. Combined with the fact that the patient received osimertinib for only 1 month, we speculated that the patient was not a single L858R mutation, but had the EGFR T263P mutation and MET amplification initially, which resulted in intrinsic resistance to osimertinib.

Meanwhile, MET amplification is a well-known mechanism of acquired resistance to EGFR-TKIs, which leads to EGFR-TKI resistance through activation of EGFR-independent ErbB3 phosphorylation and downstream activation of the PI3K/AKT pathway (6). MET and EGFR have overlapping and complementary growth and proliferation pathway activation. A phase I/II clinical study demonstrated that the combination of erlotinib and capmatinib is safe and effective and may help to treat NSCLC patients resistant to first-line therapy and increase the duration of response to TKI (7). The TATTON study also proved that combined EGFR and MET inhibition is reasonable to overcome resistance caused by simultaneous MET amplification (8). Given the patient’s financial situation and her physical condition, we finally chose erlotinib in combination with capmatinib and achieved good results, which also provides a valuable clinical reference for patients with similar compound mutations.

In this case, we report a rare EGFR mutation, EGFR T263P, a missense mutation located on ECD (domain II) that induces the conversion from Thr to Pro at position 263 encoded at EGFR exon 7. Previously, a series of missense mutations in the ECD of EGFR have been reported in cases of colorectal cancer, glioma and neuroblastoma. This is the first reported detailed clinical report of EGFR T263P mutations in patients with NSCLC. The majority of studies indicated that the T263P mutation might be sensitive to second-generation TKIs, such as afatinib, because afatinib is an irreversible ErbB family blocker(EGFR、HER-2、HER3 and HER4)that potently inhibits signaling from all ErbB family receptor homodimers and heterodimers (9, 10). Thanh et al. found that a Vietnamese NSCLC patient with the T263P mutation who received first-line afatinib treatment could achieved a mTTF of 10.8 months (11). However, Ba/F3 cells carrying the T263P variant in glioblastoma were also reported to be oncogenic and sensitive to the first-generation TKI, erlotinib (12). Similarly, the M277E mutation, which is also an EGFR extracellular region mutation, had dramatic antitumor responses to erlotinib treatment and could cause EGFR autophosphorylation in the absence of the EGFR ligand EGF, thus promoting tumor development (13).

**Figure 3:**
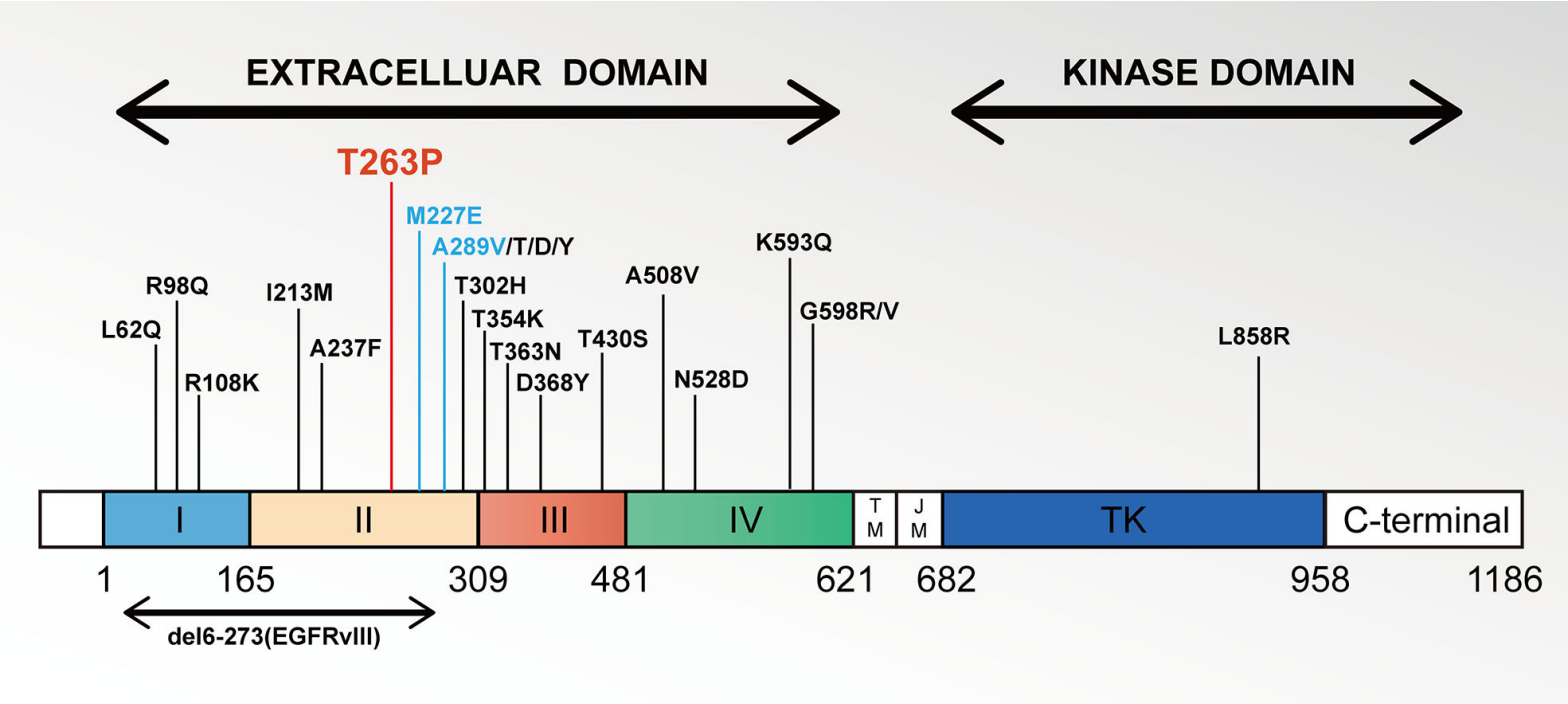
Schematic representations of EGFR gene extracellular domain mutations in NSCLC. Notes: Locations of ECD missense mutations are given within the EGFR gene found in the literature until now. Each vertical bar represents one sample harboring the indicated mutation.

According to a large retrospective study, ECD mutations account for only 0.36% (36/10100) of NSCLC and are more likely to co-occur with EGFR KD mutations, especially L858R (14). Thus, we summarized the ECD mutations that have been reported thus far in the literature on NSCLC (**Figure 3**) (15–18). The response of different ECD mutations to different EGFR-TKIs remains controversial. EGFR ECD mutations are generally considered to have a poor response to TKIs. Leilei et al. reviewed 21 patients resistant to TKI and found that one-third of NSCLC patients harbor ECD mutations (16). In contrast, some ECD mutations may also not affect the efficiency of the targeted therapy. The EGFR A289V mutation in NSCLC received the first-generation EGFR-TKIs (Icotinib) treatment and obtained the efficacy of PR for more than 5-month (19). Another patient in stage IV NSCLC with rare EGFR M277E mutation and high expression of PD-L1 had a 5 months PFS after first-line erlotinib and radiation treatment (20). Our case is the third real clinical case that demonstrates the effectiveness of EGFR ECD for first-generation TKI therapy.

In conclusion, we described a Chinese NSCLC patient with a rare EGFR T263P mutation, L858R mutation and MET amplification who achieved good efficacy with erlotinib and capmatinib, with a PFS of more than 19 months. EGFR ECD mutations, such as the T263P mutation may contributing to resistance to osimertinib. Combined with the related literature, we considered erlotinib to be efficacious for the EGFR T263P mutation with the L858R mutation, but afatinib may also be a promising choice. This finding will enlighten us to treat patients with EGFR ECD mutations and further investigate treatment strategies.

## Data availability statement

The original contributions presented in the study are included in the article/supplementary materials. Further inquiries can be directed to the corresponding author.

## Ethics statement

The studies involving human participants were reviewed and approved by the Affiliated Hospital of Nanchang University. The patients/participants provided written informed consent to participate in this study. Written informed consent was obtained from the individual(s) for the publication of any potentially identifiable images or data included in this article.

## Author contributions

(I) Conception and design: YL. (II) Administrative support: YW and XZ. (III) Provision of study materials or patients: CF and XQ. (IV) Collection and assembly of data: QW. All authors contributed to the article and approved the submitted version.

## Funding

This research was supported by grants from the National Natural Science Foundation of China (No.81560379, 81460292, 81660315), and the Surface Project of the Natural Science Foundation of Jiangxi Province (No.20181BAB205046, No.20202BAB216031).

## Conflict of interest

The authors declare that the research was conducted in the absence of any commercial or financial relationships that could be construed as a potential conflict of interest.

## Publisher’s note

All claims expressed in this article are solely those of the authors and do not necessarily represent those of their affiliated organizations, or those of the publisher, the editors and the reviewers. Any product that may be evaluated in this article, or claim that may be made by its manufacturer, is not guaranteed or endorsed by the publisher.

## Abbreviations

EGFR, Epidermal growth factor receptor; ECD, extracellular domain; TM, transmembrane; KD, intracellular kinase; TKIs, tyrosine kinase inhibitors; NSCLC, non-small cell lung cancer; PFS, progression-free survival.
